# Evaluation of the Hair Cell Regeneration in Zebrafish Larvae by Measuring and Quantifying the Startle Responses

**DOI:** 10.1155/2017/8283075

**Published:** 2017-01-29

**Authors:** Changquan Wang, Zhenmin Zhong, Peng Sun, Hanbing Zhong, Hongzhe Li, Fangyi Chen

**Affiliations:** ^1^Department of Biomedical Engineering, South University of Science and Technology of China, Guangdong, China; ^2^Research Service, VA Loma Linda Healthcare System, Loma Linda, CA 92357, USA; ^3^Department of Otolaryngology, Head & Neck Surgery, Loma Linda University School of Medicine, Loma Linda, CA 92350, USA

## Abstract

The zebrafish has become an established model organism for the study of hearing and balance systems in the past two decades. The classical approach to examine hair cells is to use dye to conduct selective staining, which shows the number and morphology of hair cells but does not reveal their function. Startle response is a behavior closely related to the auditory function of hair cells; therefore it can be used to measure the function of hair cells. In this study, we developed a device to measure the startle response of zebrafish larvae. By applying various levels of stimulus, it showed that the system can discern a 10 dB difference. The hair cell in zebrafish can regenerate after damage due to noise exposure or drug treatment. With this device, we measured the startle response of zebrafish larvae during and after drug treatment. The results show a similar trend to the classical hair cell staining method. The startle response was reduced with drug treatment and recovered after removal of the drug. Together it demonstrated the capability of this behavioral assay in evaluating the hair cell functions of fish larvae and its potential as a high-throughput screening tool for auditory-related gene and drug discovery.

## 1. Introduction

Due to its miniature size, prolific reproduction, and the external development of the transparent embryo, the zebrafish is a leading model for developmental and genetic studies, as well as in toxicology and omics-based research [1–6]. Despite being genetically more distant from humans than other models, the vertebrate zebrafish has comparable organs and tissues, such as heart, kidney, pancreas, bone, cartilage, and even hearing organs [7, 8]. Indeed, the zebrafish is nowadays an established animal model for gene and drug screening in auditory research and has become a popular model organism for the study of hearing and balance system over the past 20 years [9–12].

The zebrafish carries numerous valuable features as a model in auditory research. For instance, several dozens of hearing-related genes have been discovered in zebrafish and many of them similarly influence the inner ear of humans and other vertebrates [7, 8]. In addition, the sensitivities to a variety of ototoxins, otoprotectants, and otoregeneratives are comparable to those in the zebrafish and in humans [6, 10]. The hair cells in the lateral line system are homologous with the ones in a human's inner ear, only located superficially on zebrafish's skin, with excellent permeability of various dyes and chemicals [13]. Recent advances in studying the biophysical properties of the zebrafish hair cell provided evidence on how to relate the findings in the zebrafish hair cell to their mammalian counterpart [14, 15].

Loss of sensory hair cells is the leading cause resulting in deafness or hearing deficits, and the process is not reversible in mammalian vertebrates. There is no or very limited hair cell regeneration after hair cell damage or death. Postnatal hair cell death in humans is often induced by bacterial infections, damage from prolonged noise exposure, and treatments with certain ototoxic drugs such as aminoglycoside antibiotics or chemotherapeutic agents. In contrast to mammalian vertebrates, robust hair cell regeneration occurs in most nonmammalian vertebrates, including zebrafish [16, 17]. In combination with the advantageous technical nature of zebrafish, this animal model is positioned to become a unique research tool to study hair cell regeneration, as well as development [18]. Ongoing efforts are underway to identify regeneration specific genes and pathways that are regulated during particular stages of hair cell regeneration.

The hair cell regeneration in zebrafish is usually assessed through staining the hair cells and microscopically counting the cell number. In brief, the drug dose-dependent hair cell death can be examined with a particular ototoxin, such as neomycin, and subsequent time-lapsed cell regeneration can be investigated with borderline-hair cell death that is achieved by appropriate drug dose [17, 19].

Functional examination of zebrafish hair cell is difficult due to the lack of reliable quantification methods, compared to the electrophysiological measurement of auditory brainstem response, or otoacoustic emissions in mice. Zebrafish do harbor a rich repertoire of motor behaviors neurologically initiated by their sensory organs, either the lateral line system or the auditory system [20]. For instance, the startle response has some definitive and stable traits and can be simply triggered by a tap on the zebrafish container [21]. The startle response is intense and rapid and typically is comprised of two stages. The fish body first bends into a characteristic C-shape away from the intense stimulus within 10 msec. Afterwards, the body exhibits a small reversed curve, followed by fast swimming. The startle response can be triggered by acoustic stimuli from 5 dpf and throughout adulthood, with similar intensity threshold and frequency range [22]. These traits allow us to utilize the startle response as a behavioral tool to reliably assess hair cell damage and pertinent intervening effects. Compared to the hair cell counting method, this behavioral assay is noninvasive, so that the same fish can be examined multiple times and at various stages of the process. This system measures dozens of fish larvae each time, so that it can be used as a high-throughput drug or gene screening assay.

Deviant from previous systems for startle response measurement, significant improvement has been made to increase the accuracy. Using the system, we have successfully quantified the startle response in zebrafish (1) immediately after, (2) one day after, and (3) three days after drug exposure. The hair cells in the lateral line were also stained and counted at stages (1) and (3) to verify the damage and regeneration. The startle response results showed similar trends as what hair cell counting did but with much less effort. It demonstrated that this system can facilitate regenerative research in the zebrafish and improve and expedite our understandings in regenerative pathways and regulations in hair cell development and regeneration.

## 2. Materials and Methods

### 2.1. Animals

Wild-type TU fish line was raised and maintained in a recirculating aquaculture system according to standards described by Kimmel et al. [21]. Zebrafish larvae were maintained in embryo medium containing 0.002% Methylene Blue as a fungicide. Larvae were fed with dry food (Zeigler Bros Inc., PA, USA) starting at 5 dpf.

### 2.2. Staining and Imaging

Neomycin was used to induce damage in neuromast hair cells. It was applied to 7-dpf zebrafish larvae in the culture medium for duration of 24 hours. At the end of drug treatment, 8-dpf zebrafish were incubated in 8 *μ*M Yo-Pro-1 dye (Y3603, Molecular Probes, OR, USA) dissolved in culture medium for 1 hour at 28.5°C. After rinsing 3 times, fish were anaesthetized with 0.01% tricaine and mounted with methylcellulose in a depression slide for observation. Stained neuromasts in the lateral trunk were quantified with stereomicroscope (SMZ18, Nikon) using a 13.5x objective. For confocal imaging, fish were embedded with 1.5% low melting agarose gel. Lateral line neuromasts in the trunk region were visualized by a Leica confocal microscope TCS SP8.

### 2.3. Instrumentation for Startle Response

**Figure 1:**
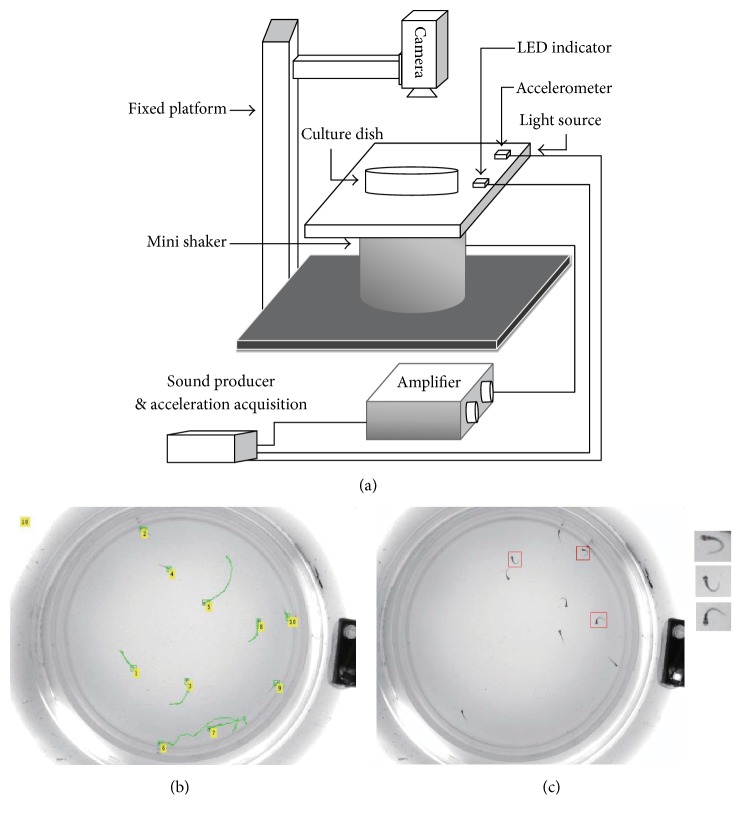
Recording the startle response in zebrafish. (a) Instrumentation for the measurement of startle response. (b) Moving traces identified from multiple picture frames after delivering a stimulus. (c) Characteristic C-bend motion identified in a single picture frame from a subset of zebrafish.

An instrument system was developed to measure the startle responses of the fish larvae. The schematic of the system was shown in Figure 1(a). The fish was contained in a Petri dish within a thin layer (2 mm) of water. This assures that every fish is within the focal range of the lens and the magnification is identical. The dish was illuminated with a light guide panel, providing evenly distributed illumination, an improvement from the previous practice with beam lighting from the side [22]. This illumination improved the image quality, resulting in better accuracy for the image processing process. The Petri dish was glued on the light guide panel with transparent glue and the light panel was glued on a mini vibrator, which generates acoustic vibrations with varying frequency and amplitude under the control of electrical signal input. The stimulus vibration is conducted to the Petri dish via the light guide panel. A MEMS-based accelerometer was also glued on the panel. This is applied to monitor the stimulus in real time. In addition, a laser Doppler vibrometer was used to measure the vibration of the water surface under various stimulations prior to the test. This step provided a direct measure of the stimulus that would be applied to the fish. Thus, stimulus parameters had been confirmed prior to the actual measurement and stimulus precision was guaranteed.

A digital camera system was mounted on a microscope frame to monitor the Petri dish and zebrafish from the top. With the transillumination, the fish larva body appears as dark region in each image frame and the fish larvae were segmented from the background with in-house software, developed within MATLAB (MathWorks, MA, USA). With the segmented fish body, the position of the fish within the Petri dish can be located. By connecting the position in each frame for a fish larva, its movement during each experiment can be extracted from the recorded video. As proposed in [22], the moving distance of the fish larvae under a short tone burst stimulus can be used as a measure of its auditory startle response.  Figure 1(b) shows the trace of 10 fish larvae after a stimulus. The mean distance of the fish can be calculated from the trace. A potentially more accurate but less sensitive measure is to count the number of fish larvae that demonstrate a C-shape motion right after the auditory stimulus. The C-shape motion is specific to the auditory startle response and lasts less than 10 ms upon stimulation [22]. The speed of the camera is 500 fps, which allows capturing the fast C-shape motion of each fish larva inside the dish. The number of fish with C-shape motion after each stimulus was calculated from several frames of the video. Both the mean distance and the number of fish with C-bend motion were calculated and used to quantify the startle responses. Here in this report we only showed the mean distance results.  Figure 1(c) shows that three fish larvae demonstrated the C-bend motion in one single frame.

### 2.4. Verification of Instrument System

To verify the efficacy of the instrument system, an experiment was performed to measure the startle responses of zebrafish larvae to sound stimulus with different intensity in fish that were treated with or without ototoxic drug. To test the relationship between the startle response and stimulus level, 400 Hz tone bursts with 3 different sound levels were applied to the amplifier to drive the vibrator. The stimulus mid-level was chosen by visually observing that more than 5 larvae (without drug treatment) showed significant movement. The high level is about 10 dB above and the low level is about 10 dB below the mid-level. For each stimulus level, 10 repeats were performed to achieve the statistical significance. Between each stimulus, 100 sec of break was applied to avoid the adaptation, as suggested in [22]. To test the sensitivity of the system to ototoxic drug, 7-dpf zebrafish larvae were treated with neomycin of 3 different levels of concentration, 0, 0.16, and 1.6 *μ*M, for 24 hours. Higher concentration of 8 *μ*M neomycin resulted in high death rate; thus it was only used in the staining experiment for hair cell survival and recovery. The startle responses were measured with the system right after rinsing the larvae at 8 dpf, with the tone burst stimulus of 400 Hz of the same sound level.

### 2.5. Recovery of Zebrafish Larvae from Drug Exposure

**Figure 2:**
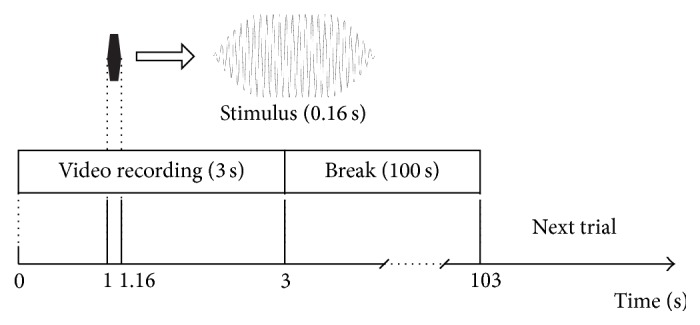
Time course for the measurement of startle response in zebrafish.

With the instrument system introduced earlier, we performed the startle responses as well as the traditional hair cell counting technique to monitor the recovery of the auditory function of the zebrafish larvae. In each test, 10 larvae were placed in the Petri dish. Two testing systems were used in parallel so that in total 20 larvae were tested for each experiment. The stimulus waveform was a tone burst of 160 ms with 30 ms rise and fall time, as shown in Figure 2. The stimulus frequency was 400 Hz and the stimulus level was 39 mm/s as the vibration velocity of the water surface. The absolute sound pressure level of this stimulus was impractical to measure due to the shallow water (~2 mm). Therefore, the vibration of the water surface at this sound level was measured by a laser Doppler interferometer to ensure the consistency.

One hundred zebrafish larvae were used in the present study. At 7 dpf, larvae were divided into three groups: control (i.e., 0) and 0.16 *μ*M and 1.6 *μ*M neomycin treatment. The startle responses of 20 larvae were tested before adding the drug. The larvae were merged in culture medium with neomycin for 24 hours and then rinsed with clean culture medium for three times. At 8 dpf, right after rinsing, 20 larvae from each group were tested with the startle response. The same test was again performed at 9 and 11 dpf (24 hrs and 72 hrs after rinsing) to monitor potential recovery due to expected hair cell regeneration.

In parallel with the startle response test, the hair cell damage by neomycin treatment was confirmed by observing and counting the hair cell with staining. As in previous test, the larvae were divided into three groups, with neomycin concentration of 0, 0.16, or 1.6 *μ*M. The hair cells on the lateral line were stained and counted at 8 dpf right after drug treatment to check the damage and at 11 dpf, 72 hrs after the treatment, to check the regeneration.

## 3. Results

### 3.1. Characterizing Startle Response

**Figure 3:**
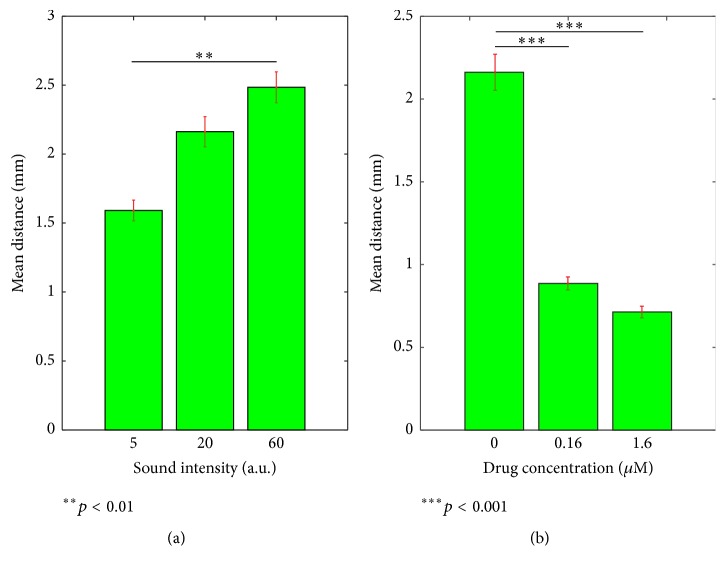
Characterizing the startle response. (a) Mean moving distance of larvae with 400 Hz sound of intensity linearly grown from 5 to 60 with an arbitrary unit. This results in a sound level in about 20 dB. The values are 1.59 ± 0.23, 2.16 ± 0.34, and 2.48 ± 0.35. There is statistically significant difference between the 1st column and 3rd column (*p* < 0.01) but not between adjacent columns. (b) Mean moving distance as function of the neomycin concentration. The values are 2.16 ± 0.34, 0.89 ± 0.12, and 0.71 ± 0.11. There is statistically significant difference between the 1st column and 2nd column (*p* < 0.001) but not between 2nd and 3rd columns (*p* = 0.09). The error bar is standard error.

We quantified the startle response by zebrafish larvae's moving distance upon sound stimulation.  Figure 3(a) shows that the mean moving distance increased with rising sound levels, in a range of 20 dB, that is, 10-fold.  Figure 3(b) shows the startle responses versus ototoxic drug concentration. The concentration of neomycin was at 0, 0.16 *μ*M, or 1.6 *μ*M. The sound stimulus was 400 Hz tone bursts.

### 3.2. Regeneration of Neuromast Hair Cells after Neomycin-Induced Hair Cell Damage

**Figure 4:**
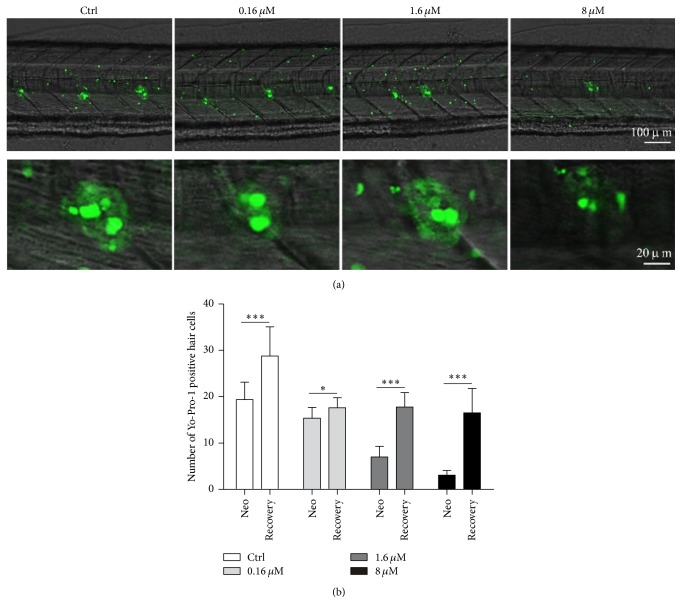
Neomycin-induced neuromast hair cell damage and regeneration. (a) Confocal image of lateral line neuromasts under neomycin treatment in wild-type zebrafish. (b) Average number of neuromast hair cells in each group. Each group consists of 10 7 dpf zebrafish larvae treated with respective concentration for 24 h and then allowed to recover for 72 h to assess hair cell regeneration. All neomycin-treated larvae showed decreased number of hair cells to some extent; statistical analyses were performed using Student's* t*-test (^*∗*^*p* < 0.05; ^*∗∗∗*^*p*  < 0.001). Error bars are standard deviation.

Previous studies have mostly demonstrated that neomycin exposure ablated hair cells in the lateral line in a dose-dependent manner [10, 16]. Here in this study, Yo-Pro-1 was used to identify hair cells from posterior neuromasts in the lateral line. After 24-hour neomycin treatment, neuromasts in the trunk region dorsal to the pelvic fin were observed and hair cells were counted.  Figure 4(a) illustrates that a high dose of 8 *μ*M neomycin led to loss of most hair cells; the Yo-Pro-1 positive residues were random and dispersed, unlike the cluster-like organization observed with lower neomycin dosing. Compared to the control group, a smaller and less number of hair cells were observed with either 0.16 or 1.6 *μ*M neomycin treatment (Figure 4(b)). Three-day recovery enabled robust regeneration of hair cells (Figure 4(b)), which is consistent with previous reports investigating the precursor pool maintenance in lateral line hair cells [23].

### 3.3. Startle Responses of the Same Procedure

**Figure 5:**
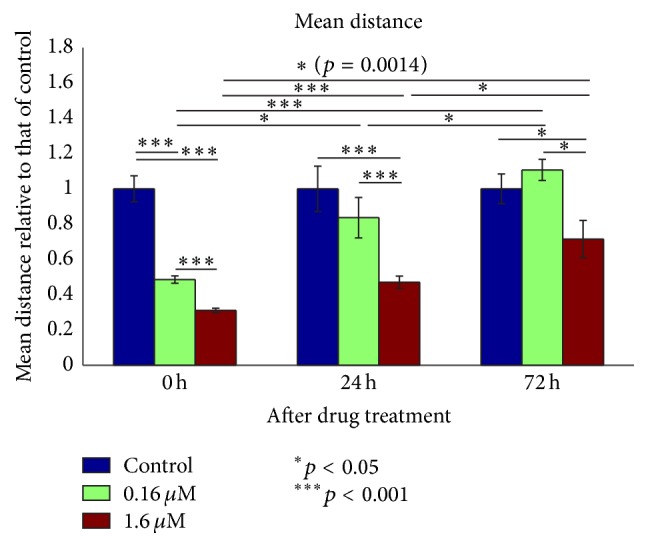
Startle response of fish larvae with neomycin treatment of different level of concentration and subsequent recovery period after drug removal. Mean moving distance after sound stimulation was used as the quantification parameter. To eliminate the variation of different days, the values are scaled by that of the control group in each day. The value in each column from left to right is 1 ± 0.23, 0.48 ± 0.06, 0.31 ± 0.03, 1 ± 0.41, 0.84 ± 0.36, 0.47 ± 0.11, 1 ± 0.27, 1.10 ± 0.19, or 0.72 ± 0.34 in the format of mean ± standard deviation. The unpaired* t*-test on adjacent columns in each day is shown to be mostly significant, except for between control and 0.16 *μ*M at 24 h or at 72 h. For the same drug concentration (e.g., 0.16 *μ*M at 0 h, 24 h, and 72 h), unpaired* t*-test shows significant (*p* < 0.05) difference between days for both 0.16 and 1.6 *μ*M cases.

Using the same experimental condition with neomycin treatment, we also evaluated the startle response with our in-house instrument system. The tone burst attributes were the same as previously described and the tone frequency was 400 Hz. The startle responses were checked 24 hrs and 72 hrs after the drug treatment. The mean moving distance of the control group is used as a reference at each checkpoint. The responses of the drug treatment groups were normalized by that of the control group to eliminate the possible variation in startle responses between different days. At 8 dpf (0 h in Figure 5), the responses of the drug treatment group are significantly smaller than that of the control. With drug treatment, the startle responses at 24 h and 72 h showed gradual growth of moving distance, compared to that at 0 h, indicating time-lapsed functional recovery.

## 4. Discussion

### 4.1. Efficacy of Using the Vibrator

In this study, a mini shaker was used as the driver to deliver acoustic vibration to the Petri dish and produce the sound stimulus to fish larvae. Although this is not a direct sound generation, it is an effective way of delivering sound stimulus. Using a load speaker in air is not efficient because of the air-water interface, where 95% of sound energy is reflected back. An aquatic speaker can be used underwater but it is not practical in this setup because the water level is only a few millimeters inside the Petri dish. The mini shaker was previously used in [24] and was experimentally effective.

### 4.2. Interference between Fish

In one of the previous systems [24], the fish larvae were placed in a multiwell plate. The design made it easier to identify each individual fish during image processing. However, the setup resulted in the uneven sound pressure level of stimulus in each well, causing inaccuracy in data collection. In the present setup, all the fish were placed in the same Petri dish. Due to the shallow water level, the sound level is evenly distributed and thus stimulus to each fish is identical. One concern on this setup is that fish larvae can sense each other in this setup without the segregation by the individual well wall. Theoretically, some fish may move after seeing others' quick motion. Yet, we doubt that visual cue contributes to the measured startle response and contaminates our data collection. In our setup, the fish are mostly distanced (see Figure 1(c)), which largely reduced the visual interference among them. In addition, if a C-bend motion was triggered by a visual cue on other fish's startle response, the latency of this motion would be extended, causing desynchronized “startle responses” among fish. However, this desynchronization was not observed. The concern on interanimal interference can also be further evaluated by using infrared illumination during the experiment [25].

### 4.3. Behavioral Test Sensitivity Based on the Startle Response

With the experimental protocol in the present study, the test sensitivity was comparable between the morphological hair cell counting method in Figure 4 and the behavioral method testing the startle response in Figure 5. Both methods were able to detect the ototoxic neomycin caused damage with the lowest tested concentration (0.16 *μ*M). With higher neomycin concentration, extended hair cell loss was observed and so was the further shortened swimming distance after the startle stimuli. Although the behavioral test produced satisfactory outcome, we believe the test sensitivity is likely further improved with modification in experiment design. For instance, prepulse inhibition was shown to increase the sensitivity by about 40 dB in a startle response test system [26], while sound pressure level of 60 dB above the hearing threshold is required to directly induce the startle response.

## 5. Conclusion

In this study, we developed a behavioral assay to evaluate the auditory function of hair cells by measuring the startle response of zebrafish larvae. By applying various level of stimulus, results showed that the system can discern a 10 dB sound level difference. Using the system, we investigated the hair cell damage and regeneration in the lateral line neuromasts of zebrafish larvae. The result from this system shows similar trend to the traditional hair cell counting methods. The startle response was reduced with neomycin treatment and recovered with hair cell regeneration. These results demonstrated the capability of this behavioral assay in evaluating the hair cell functions of zebrafish larvae and its potential as a high-throughput screening tool for auditory-related gene and drug discovery.
